# Teacher professional identity on work engagement: the moderating roles of ego-resilience and perceived organizational support

**DOI:** 10.3389/fpsyg.2025.1657911

**Published:** 2025-11-27

**Authors:** Xiajun Yu, Zihuan Xiong, Weijian Li, Hongwei Zhang, Xinwei Li, Weilong Xiao

**Affiliations:** 1 College of Education, Huzhou University, Huzhou, China; 2 School of Psychology, Zhejiang Normal University, Jinhua, China; 3 Dongfang Experimental School, Zibo, China; 4 School of Teacher Education, Zhejiang Normal University, Jinhua, China

**Keywords:** teacher professional identity, ego-resilience, perceived organizational support, work engagement, Chinese teachers

## Abstract

**Introduction:**

Few studies explored the effects of combining different resources on work behavior. This study tested the interaction effect of personal (i.e., ego-resilience) and organizational resources (i.e., perceived organizational support) in the relationship between teacher professional identity and work engagement.

**Methods:**

A total of 2,956 teachers took part in this study. Four scales measured the professional identity, ego-resilience, perceived organizational support, and work engagement.

**Results:**

Results showed that: (1) teacher professional identity has significant correlations to ego-resilience (*r* = 0.49, *p* < 0.01), perceived organizational support (*r* = 0.50, *p* < 0.01), and work engagement (*r* = 0.64, *p* < 0.01); (2) when the scores of ego-resilience and perceived organizational support were both high, the predictive effect of teacher professional identity on work engagement was the biggest (*B* = 0.72, SE = 0.07, *p* = 0.000, 95% CI = 0.58 to 0.86).

**Discussion:**

Enhancing teachers’ ego-resilience and improving organizational support are of great significance for increasing teachers’ work engagement.

## 1 Introduction

Work engagement is a positive, fulfilling, and emotionally motivating state (Bakker and Albrecht, 2018) and is characterized by energy, dedication, and focus (Schaufeli et al., 2002). Work engagement and burnout both belong to the field of professional mental health; however, the state described by work engagement is the opposite of burnout (Qi and Wu, 2018; Xiao et al., 2024; Xue et al., 2024). Initial research mainly focuses on how to alleviate the effects of work stress on job burnout, and with the emergence of positive psychology, more and more studies pay attention to how to increase employees’ work engagement (Bakker et al., 2010; Freudenberger, 2010; Li et al., 2025; Midje et al., 2024; Wu et al., 2010). This study explored the effects of teacher professional identity, ego-resilience, and perceived organizational support on teacher work engagement at the personal level and organizational level.

Teacher professional identity, which is defined as the positive cognition and behavioral tendency of teachers toward the profession, is a multi-dimensional concept comprising professional values, role values, professional belonging, and professional behavioral tendencies (Sun et al., 2025; Wei et al., 2013). As the basic motivation of teacher behavior, teacher professional identity is positively related to teacher mental health (Sun et al., 2025; Wei and Song, 2012) and career development (Qin and Liu, 2023). The Job Demands-Resources Model (JD-R Model) is used to explain the effects of job demands and resources on professional behavior (Bakker and Demerouti, 2017). Job resources refer to the working factors that can provide support and assistance to workers, which have the potential for motivation, and can promote a high level of work commitment and excellent job performance (Bakker et al., 2010). In brief, job resources refer to those resources that assist workers in achieving their work goals and that can alleviate the physical and mental strain caused by work demands. Teacher professional identity can help teachers cope with stress effectively, promote the growth of teachers, and thus be regarded as a work resource, and improve teachers’ work engagement. Furthermore, studies on other occupations have also found that there is a positive correlation between professional identity and work engagement (Um and Bardhoshi, 2025; Zhang et al., 2023). For example, the research conducted by Zhang et al. (2023) found that professional identity was positively correlated with work engagement among nurses. Based on these empirical results and the related theory, this study hypothesized that teacher work engagement can be positively related to professional identity (H1).

Ego-resilience has been defined as the manifestation of positive outcomes in the face of some form of adversity (Luthar et al., 2000; Masten, 2001). One research study conducted by Kim and Han (2021) found that ego-resilience had a moderating effect in the relationship between emotional labor and job satisfaction among nurses. Job resources can be divided into personal and organizational resources according to the main body of resources. Personal resources include physical, mental, emotional, and intellectual aspects (Hobfoll, 2002). Previous research mainly focuses on the effect of mental resources on professional behavior (Ghafoori et al., 2024; Hasani, 2024; Xanthopoulou et al., 2008). Among these resources, mental resources include personal optimism (Zhang et al., 2020), self-efficacy (Li et al., 2024), and ego-resilience. The research conducted by Li et al. (2024) found that self-efficacy can moderate the relationship between teacher professional identity and work engagement. In actual educational settings, regardless of how high their level of professional identity is, teachers are also exposed to practical obstacles (e.g., unmotivated students and problems with colleagues), which can affect their work engagement (Llorens-Gumbau and Salanova-Soria, 2014). When dealing with these issues, the teacher’s ego-resilience plays a crucial role. Teachers with high ego-resilience can learn from adversity, find meaning, and experience positive emotions. They can actively seek social support, thereby quickly replenishing the psychological resources that have been depleted (Scott et al., 2024).

Perceived organizational support refers to employees’ perceptions of the extent to which an organization values their contributions and cares about their well-being (Rhoades and Eisenberger, 2002). Research has found that perceived organizational support has a positive effect on employee outcomes (Gavino et al., 2012; Tabak and Hendy, 2016). The research conducted by Tabak and Hendy (2016) found that perceived organizational support promotes employees’ work engagement. The positive effect of organizational support on work engagement can be explained by the Theory of Social Support, which posits that perceived support from friends, colleagues, and relatives can buffer the negative influence of job stress on burnout by providing individuals with instrumental support (e.g., the way to cope with stress effectively) and emotional support (e.g., encouragement) (Cohen and Wills, 1985). Thus, when teachers perceived a high level of organizational support, they would like to put more engagement into their job.

**FIGURE 1:**
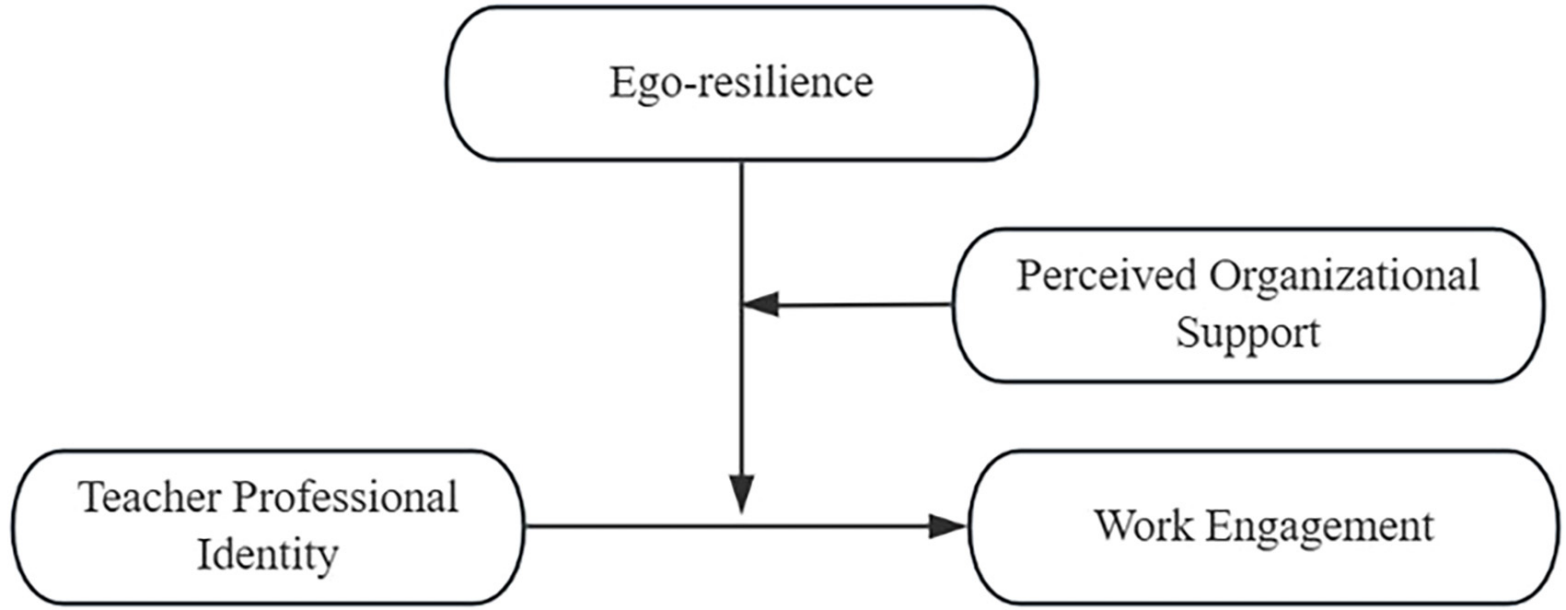
Hypothesized model.

It can be known from the summary of previous studies that ego-resilience and perceived organizational support can positively affect work engagement. Work engagement is influenced by the combined effects of personal and organizational resources (Hobfoll, 2002). According to the Job Demands-Resources model, job resources can be categorized into personal resources and organizational resources, both have a positive effect on work engagement. When both personal and organizational resources are present, they create a robust system of motivation and protection for employees, enabling them to maintain high levels of energy and work engagement. Meanwhile, related research found that when scores of self-efficacy and perceived organizational support were high, the predictive effect of teacher professional identity on teacher burnout was the biggest (Li et al., 2024). Therefore, this study hypothesized that when these two types of resources interact, the impact of teacher professional identity on work engagement is the biggest (H2). The hypothesized model can be seen in Figure 1.

## 2 Materials and methods

### 2.1 Participants and procedures

This study was conducted according to the Declaration of Helsinki and APA ethical standards and was approved by the local committee (No ZSRT2022020). Convenience sampling was used in this study. Data was collected by a platform called Credamo. The specific procedure was as follows: Firstly, we will create questionnaires on Credamo and generate a link that will be used to send to our participants. Secondly, they must finish the informed consent before filling out the questionnaire. Thirdly, they were asked to complete the questionnaires under the written instructions, and their data were finally exported to SPSS.

All participants were from Zhejiang Province. A total of 2,956 teachers took part in this study. After the initial review, 233 participants were excluded from further analysis due to missing key variables. Thus, data from 2723 teachers (valid response rate: 92.12%) were used for additional correlational and mediation analyses. Among these participants, 800 were male (29.40%). Their average age was 38.40 years (SD = 9.01), and their average seniority was 18.62 years (SD = 11.12).

### 2.2 Materials and measurements

Teacher professional identity was measured using the Chinese version of the Teacher Professional Identity Scale, compiled by Wei et al. (2013). This scale consists of four sub-dimensions: Occupational values, role values, sense of occupational belonging, and professional behavior inclination, comprising a total of 18 items. Scores ranged from 1 (strongly disagree) to 5 (strongly agree), with a Cronbach’s alpha of 0.95.

#### 2.2.1 Ego-resiliency

It was measured by the scale designed by Block and Kremen (1996). It consists of 14 items (e.g., I would be willing to describe myself as a pretty “strong” personality). Items were rated on a four-point Likert scale (1 = does not apply at all, 5 = applies very strongly). The Cronbach α of this scale was 0.93.

#### 2.2.2 Perceived organizational support

This was measured using the Perceived Organizational Support Scale designed by Eisenberger et al. (2001). In this study, we used the Chinese version that was translated by Liu et al. (2013). The scale includes eight items, and every item was scored from 1 (strongly disagree) to 7 (strongly agree). The Cronbach α was 0.89 in our study.

#### 2.2.3 Work engagement

This was measured using the Utrecht Work Engagement Scale (UWES-9) developed by Schaufeli and Bakker (2004), which consists of three dimensions (i.e., vigor, dedication, and absorption). This scale was translated into Chinese by Zhang and Gan (2005). The scale includes nine items, and every item was scored from 1 (never) to 5 (always). The Cronbach α was 0.94 in our study.

## 3 Results

### 3.1 Common method bias

Harman’s single-factor test was used to test possible common method deviation in this study. Results showed that there were 7 characteristic roots greater than 1, and the total variance was 39.89%, less than 40%, indicating that there is no obvious common methodological bias in this study (Podsakoff et al., 2003).

### 3.2 Correlation results

The results of correlation among the main variables showed that teacher professional identity positively related to ego-resilience (*r* = 0.49, *p* < 0.01), perceived organizational support (*r* = 0.50, *p* < 0.01), and work engagement (*r* = 0.64, *p* < 0.01); Ego-resilience positively related to perceived organizational support (*r* = 0.47, *p* < 0.01), and work engagement (*r* = 0.63, *p* < 0.01); Perceived organizational support positively related to work engagement (*r* = 0.54, *p* < 0.01). The specific results can be seen in Table 1.

**TABLE 1:** Correlational analyses of main variables.

Variables	*M*	SD	1	2	3	4
TPI	4.43	0.58	–			
ER	3.15	0.56	0.49**	–		
POS	3.78	0.82	0.50**	0.47**	–	
WE	3.87	0.80	0.64**	0.63**	0.54**	–

TPI, teacher professional identity; ER, ego-resilience; POR, perceived organizational support; WE, work engagement.

**p < 0.01.

### 3.3 Results of moderators of ego-resilience and perceived organizational support

Model 3 (Process macro in SPSS 25.0, Hayes, 2013) was used to test the moderating roles of ego-resilience and perceived organizational support in the relationship between teacher professional identity and work engagement. Results showed that the interaction effect of teacher professional identity, ego-resilience, and perceived organizational support on work engagement was significant (*B* = 0.12, SE = 0.04, *p* = 0.001, 95% CI = 0.05 to 0.19). Further simple analysis showed that when scores of ego-resilience and perceived organizational support were high, the effect of teacher professional identity on work engagement was the biggest (*B* = 0.72, SE = 0.07, *p* = 0.000, 95% CI = 0.58 to 0.86). The specific results are shown in Table 2.

**TABLE 2:** Results of moderating roles of ego-resilience and perceived organizational support.

	Y: WE				
Variables	*B*	SE	*P*	95% CI	
X: TPI	0.52	0.02	0.000	0.48, 0.57	
M: ER	0.48	0.02	0.000	0.43, 0.53	
W: POS	0.17	0.02	0.000	0.13, 0.20	
X × M	0.17	0.04	0.000	0.08, 0.25	
X × W	−0.002	0.03	0.952	−0.06, 0.06	
M × W	−0.001	0.03	.974	−0.05, 0.05	
X × M × W	0.12	0.04	0.001	0.05, 0.19	
Constant	3.84	0.01	0.000	3.82, 3.87	
			**Conditional indirect of X on Y**		
		***B***	**SE**	***P***	**95%CI**
M: M−1SD	W: M−1SD	0.49	0.03	0.000	0.44, 0.54
	W: M+1SD	0.38	0.06	0.000	0.27, 0.49
M: M−1SD	W: M−1SD	0.58	0.05	0.000	0.49, 0.67
	W: M+1SD	0.72	0.07	0.000	0.58, 0.86

TPI, teacher professional identity; ER, ego-resilience; POR, perceived organizational support; WE, work engagement.

## 4 Discussion

This study tested the effects of teacher professional identity, ego-resilience, and perceived organizational support on work engagement. Results showed that work engagement was positively predicted by teacher professional identity, supporting H1. This result was similar to other previous studies (Li et al., 2024, 2025; Sun et al., 2025). For example, the research conducted by Li et al. (2024) found that work engagement was positively related to teacher professional identity. This result could be explained by JD-R theory, which posits that job resources can promote a high level of work commitment and excellent job performance due to job resources have the nature of motivation (Bakker et al., 2010). Teacher professional identity, as the positive cognition and behavioral tendency of teachers toward the profession, has the most basic motivation to work, and thus can promote teacher work engagement.

Meanwhile, this study found that when teachers have a high level of ego-resilience and perceived organizational support, the predictive effect of teacher professional identity on work engagement was the strongest, supporting H2. This result, on the one hand, indicates that job resources can promote work engagement (Qi and Wu, 2018). On the other hand, this result suggests that when different kinds of resources work together, they may have the strongest effect. This result was similar to the research conducted by Sun et al. (2025), who found that when competence and growth values, and ego-resilience were both high, the predictive effect of teacher professional identity on teacher empathy was strongest. Furthermore, we did an additional analysis of when ego-resilience and perceived organizational support exchange positions of their moderating effects, and results showed that the triple interaction effect of teacher professional identity, ego-resilience, and perceived organizational support on work engagement was not significant. This result indicates that in the relationship where teacher professional identity influences work engagement, the different resources exert their effects at different levels and under different conditions. Specifically speaking, perceived organizational support may be a more fundamental boundary condition for the motivational roles of teacher professional identity and ego-resilience. In environments lacking sufficient organizational support, even if teachers have a strong sense of professional identity and ego-resilience, their level of work engagement may still be difficult to enhance (Xu et al., 2023, 2025).

This study has some theoretical and practical implications. Theoretically, this study supported the JD-R model and expanded this theory to some degree. JD-R theory describes the different effects of job demands and resources on professional health, and it introduces the related resources and demands (Bakker and Demerouti, 2017). However, this theory did not explore the interaction effect of different kinds of resources. This study examined the interaction effect of personal (i.e., ego-resilience) and organizational resources (i.e., perceived organizational support) on teacher work engagement, finding that when both ego-resilience and organizational support were high, the effect of teacher professional identity on work engagement was the most pronounced. Practically, this study indicated the importance of teacher professional identity, ego-resilience, and perceived organizational support on work engagement. For school managers, trying their best to provide support to teachers can help teachers engage with their work. Meanwhile, through relevant training to enhance teachers’ professional identity and ego-resilience, only in this way can both individuals and organizations play their roles in teachers’ work engagement.

Some limitations should be noted in the study. First, the cross-sectional nature of data is a key limitation, as it prevents us from drawing causal inferences. Longitudinal or experimental designs should be used to better establish the causal directions among these variables in future research. Second, using a single self-report questionnaire for all measures carries a risk of Common Method Variance. Future studies can mitigate this by using multi-source data (e.g., supervisor ratings of work engagement) or incorporating temporal separations in the measurement of predictors and outcomes. Third, the sample was drawn from a single province (i.e., Zhejiang) in China, which may limit the generalizability of findings. Future research should replicate this study in other regions of China with different developmental levels and in cross-national contexts to enhance its external validity. Fourth, some variables (e.g., student behavior) may influence the findings, with future studies to include a broader set of variables, particularly key job demands from the JD-R model, to gain a more comprehensive understanding of the antecedents of teacher work engagement and to control for their potential confounding effects.

## Data Availability

The data can be obtained from the corresponding author with reasonable request.
